# Method-comparison study between a watch-like sensor and a cuff-based device for 24-h ambulatory blood pressure monitoring

**DOI:** 10.1038/s41598-023-33205-z

**Published:** 2023-04-15

**Authors:** Martin Proença, Jeremias Ambühl, Guillaume Bonnier, Théo A. Meister, Jérémy Valentin, Rodrigo Soria, Damien Ferrario, Mathieu Lemay, Emrush Rexhaj

**Affiliations:** 1 Signal Processing Group, Swiss Center for Electronics and Microtechnology (CSEM), Neuchâtel, Switzerland; 2 Department of Cardiology and Biomedical Research, University Hospital, Bern, Switzerland

**Keywords:** Public health, Cardiovascular diseases, Hypertension

## Abstract

The use of 24-h ambulatory blood pressure monitoring (ABPM) has been continuously increasing over the last decades. However, cuff-based devices may cause discomfort, particularly at night, leading to potentially non-representative blood pressure (BP) values. We investigated the feasibility of a cuff-less BP monitoring solution in 67 subjects undergoing conventional 24-h ABPM. A watch-like optical sensor was attached at the upper arm or wrist at the contralateral side of the cuff. Systolic (SBP) and diastolic BP (DBP) values were estimated from the measured optical signals by pulse wave analysis. Average 24-h, daytime and nighttime BP values were compared between the conventional monitor and the cuff-less sensor. The differences between both methods—expressed as mean ± standard deviation (95% limits of agreement)—were of − 1.8 ± 6.2 mmHg (− 13.9, 10.3) on SBP and − 2.3 ± 5.4 mmHg (− 13.0, 8.3) on DBP for 24-h averages, of − 1.5 ± 6.6 mmHg (− 14.4, 11.4) on SBP and − 1.8 ± 5.9 mmHg (− 13.4, 9.9) on DBP for daytime averages, and of 0.4 ± 7.5 mmHg (− 14.4, 15.1) on SBP and − 1.3 ± 6.8 mmHg (− 14.7, 12.0) on DBP for nighttime averages. These results encouragingly suggest that cuff-less 24-h ABPM may soon become a clinical possibility.

## Introduction

The use of 24-h ambulatory blood pressure monitoring (ABPM) has been continuously increasing over the last decades for the formal diagnosis of hypertension. Especially indicated in cases of suspected white-coat effect, masked, or nocturnal hypertension, 24-h blood pressure (BP) profiles are stronger predictors of cardiovascular morbidity and mortality than office or home BP monitoring^1^. However, ABPM also suffers from several limitations in general practice, such as its limited availability, the discomfort it may cause, the reluctance of some patients to repeat measurements, or the occasional inaccuracy of readings^2^. In particular, ABPM devices tend to reduce sleep time and the inflation of the cuff can cause arousal from sleep, thereby affecting the BP itself and leading to non-representative dipping and nighttime values^3,4^. A cuff-less alternative BP device to a conventional ABPM one, would be a significant leap forward in this matter.

Therefore, in the present study, we investigated a cuff-less watch-like optical sensor to estimate 24-h BP values. The sensor uses photoplethysmography (PPG) signals, an optical technique for measuring changes in blood volume in superficial arteries, and already widely deployed in pulse oximeters for oxygen saturation monitoring, or more recently in smartwatches for the measurement of heart rate (HR)^5^. Over the last decade, an increasing number of studies have explored the use of PPG technology for the non-invasive estimation of blood pressure (BP) through so-called pulse wave analysis (PWA) techniques^6^. By analyzing the morphology of PPG waveforms—affected by BP wave transmission and reflection along the arterial tree—physiological features representative of arterial distensibility, and thereby indirectly of BP, can be extracted algorithmically and mapped to values of BP through dedicated physiological models. The purpose of the present study was to investigate the feasibility of using a cuff-less watch-like PPG sensor for 24-h ABPM by comparing the PPG-derived BP estimates with the ABPM values estimated by a conventional oscillometric device.

## Methods

This study was conducted in accordance with the amended Declaration of Helsinki. The protocol was approved by the Ethical Committee of the Canton of Bern (Kantonale Ethikkommission, Switzerland, application 2016-00614), and registered at ClinicalTrials.gov (NCT04119518, date of registration: 08/10/2019). All participants provided written informed consent.

### Participant recruitment

**Figure 1:**
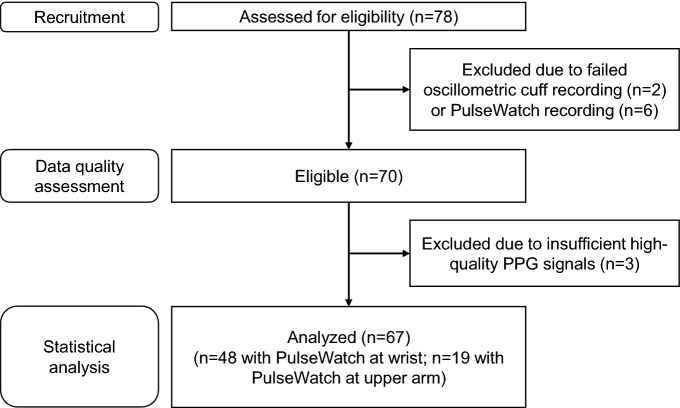
Flow diagram of the study, from participant recruitment to statistical analysis.

78 patients were recruited (see Fig. 1) in the outpatient and inpatient clinic of the Department of Cardiology, Inselspital Bern, Switzerland, between January 2019 and June 2021. Patients who were treated for arterial hypertension, patients who were screened for arterial hypertension and normotensive subjects that met the inclusion and none of the exclusion criteria (see Supplementary Information) were enrolled in the study.

### Data acquisition

**Figure 2:**
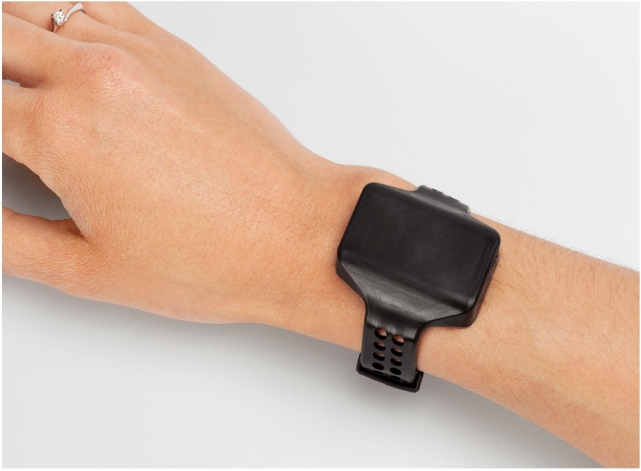
Cuff-less watch-like device used in the present study (PulseWatch, CSEM, Neuchâtel, Switzerland), which contains a photoplethysmographic (optical) sensor and an accelerometer, and can be attached at the wrist or the upper arm.

The participants were equipped at the non-dominant arm with a standard oscillometric device (Spacelabs 90227 OnTrak Ambulatory Blood Pressure monitor, Spacelabs Healthcare, Washington, USA) validated for 24-h ABPM^7^. Bladder cuff size was adapted to the arm circumference of patient and positioned at the level of heart, according to ESC guidelines on BP measurement^8^. At the contralateral wrist or upper arm (this was changed during the study because of lower PPG sensitivity to arm motion at the upper arm), the participants were wearing a watch-like device (PulseWatch, CSEM, Neuchâtel, Switzerland) (see Fig. 2) containing a PPG sensor and a 3-axis accelerometer. When worn at the upper arm, the device was attached using a gauze bandage. Data acquisition was started roughly at the same time with both devices; a time alignment procedure was performed in post-processing to ensure adequate synchronization between the oscillometric cuff and the PulseWatch (see PPG data processing and BP estimation section). Data acquisition was stopped upon return of the participant to the doctor’s office, approximately 24 h later. Oscillometric BP cuff measurements were performed every 20 min during daytime, and once per hour at night. The awake and sleeping time were defined according to the participants’ written information. PPG and accelerometer data were recorded continuously at a sampling rate of 100 Hz by the PulseWatch.

### PPG data processing and BP estimation

**Figure 3:**
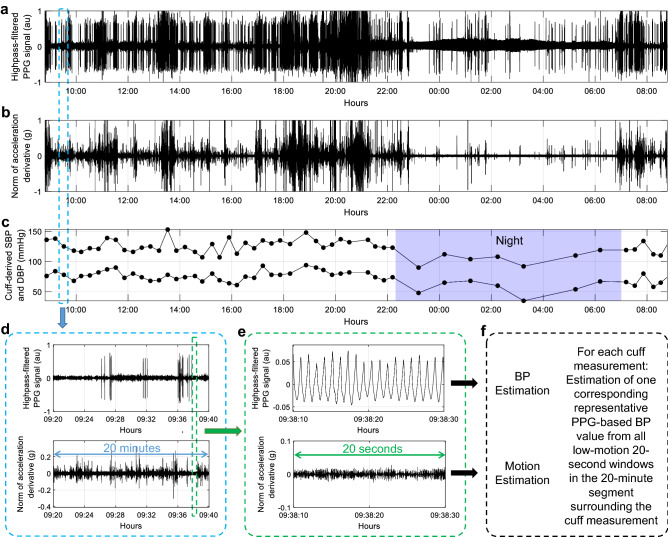
Illustration of the PPG data processing approach, from signal segmentation to BP estimation. (**a**) Example of a 24-h baseline-corrected (highpass-filtered) PPG signal measured by the PulseWatch. (**b**) The corresponding 24-h first derivative of the acceleration Euclidian norm used for assessing the amount of motion. (**c**) The 24-h cuff-based BP profile of the patient. (**d**) 20-min segment surrounding a cuff measurement. (**e**) 20-s window used to obtain one BP estimate and one motion estimate. (**f**) From the BP estimates of all low-motion 20-s windows in the 20-min segment, one representative PPG-based BP value is estimated. *PPG* photoplethysmography, *BP* blood pressure, *au* arbitrary units.

All data were post-processed and analyzed using Matlab (The MathWorks, Inc, Natick, MA, USA), version R2022a. For each participant, the synchronization between the cuff and the PulseWatch recordings was ensured by cross-correlation of the HR estimated by both devices. The overall data processing method is illustrated in Fig. 3, and detailed hereafter. The PulseWatch’s PPG and accelerometer signals were analyzed using a 20-s moving window of analysis with an 18-s overlap. In other words, every 2 s, the surrounding 20 s of PPG and accelerometer signals were used to obtain an estimated BP value and an estimated amount of motion, respectively. The BP estimate was obtained using our PWA algorithm^9^ on the PPG signals, as previously detailed^10,11^. No user demographics are needed to estimate the BP. The amount of motion for each 20-s segment was estimated using the Euclidian norm of the 3-axis accelerometer data. 20-s segments for which the amount of motion was considered too high were discarded. A 20-s segment was considered as having high motion if the first derivative of the Euclidian norm of its acceleration exceeded 50 mg for more than 1% of the time. The PWA algorithm systematically evaluates the quality of the PPG signals when trying to extract physiological features from their waveforms. 20-s segments not discarded due to high motion but still considered as unreliable by the PWA algorithm (for instance due to excessive noise in the waveform) were also discarded. As a last step, for a meaningful comparison between the cuff-based and the PPG-based BP estimates, each oscillometric cuff measurement was paired with a representative BP value of the PPG-based estimates falling within ± 10 min around that cuff measurement. Each representative BP value was obtained by discarding outliers among the PPG-based estimates using the median absolute deviation^12^ and averaging the remaining inliers. In 3 recordings, after rejection of high-motion segments and segments with low-quality PPG signals, an insufficient amount of analyzable data remained. These recordings were therefore rejected prior to statistical analysis (see bottom part of Fig. 1). The practice guidelines of the European Society of Hypertension for ABPM^2^ recommend repeating an ABPM session if less than 70% of the 24-h cuff readings are successful, or if less than 20 daytime or 7 nighttime cuff readings are successful. Because we only performed cuff measurements on an hourly basis during the night to minimize patient discomfort, 27 out of our 67 recordings (40%) did not fulfill these recommendations, mainly regarding the minimum of 7 nighttime cuff readings. Nevertheless, for the present method-comparison study, whose primary aim was a feasibility evaluation rather than a proper validation of a novel cuff-less approach, we chose to keep these recordings in our analysis.

The BP values estimated from the PPG signals by the PWA algorithm are expressed in pressure units (mmHg) but are centered around an arbitrary offset value that needs to be adjusted through a calibration procedure. In an ABPM context, setting the value of this initial offset can be done at the beginning of the ABPM session, using a cuff measurement while the patient is still at the doctor’s office. In practice, this not only requires the cuff measurement used for this calibration procedure to be as exact as possible to avoid passing the cuff’s inaccuracy on to the PPG-based estimate, but it also requires ensuring that the PPG signal quality is sufficient during the procedure. However, in the present study, because the PPG data were recorded in the PulseWatch and only post-processed offline, there was no way to guarantee sufficient PPG signal quality in real-time during the patient’s visit, and therefore no way to ensure the possibility to properly adjust the offset of the PPG-based BP estimate. Instead, we had to perform this offset adjustment procedure during offline post-processing. To that end, we mimicked a calibration procedure as we would imagine it being done at the doctor’s office, by using high-quality PPG measurements and their respective cuff BP values. For each patient, we used the six daytime measurements where the PPG signal quality was the highest and calculated the calibration offset as the average difference between the six PPG-derived BP measurements and their respective cuff-derived BP values. The rationale for the use of six measurements is a trade-off between what seems as a reasonably acceptable amount of consecutive cuff measurements to perform for a patient at the doctor’s office, and the need for minimizing the cuff-induced bias in the calibration procedure. Indeed, by repeating and averaging N measurements, the standard deviation (SD) of the cuff error is reduced by a factor √N. During statistical analysis (see next section), the six daytime measurements used for calibration for each subject were removed from the analysis as they would otherwise artificially increase the agreement between the PPG-based and cuff-based BP measurements.

It is important to note that any left-to-right arm BP difference does not have any influence in our study when comparing the PPG-based and cuff-based BP measurements, despite both devices being placed at different arms, as any potential difference is compensated for by the calibration procedure.

### Statistical analysis

The agreement between the cuff-based and the PPG-based BP 24-h, daytime and nighttime averages was assessed through Bland–Altman analysis^13^. To that end, the cohort-wise mean and standard deviation of the differences between both devices were assessed, along with their 95% limits of agreement. Considering the cuff-based measurements as reference, the bias thus represents the accuracy of the PPG-based measurement, and the SD its precision. Because it is standard practice to provide mean BP (MBP), pulse pressure (PP), and HR values in an ABPM clinical report, we also reported the accuracy and precision figures obtained for these three variables. Another useful ABPM variable—despite its poor reproducibility^14^—is the nocturnal BP decline (dipping), calculated as (1 − nighttime average/daytime average) and expressed in %. We evaluated the concordance rate (CR) of our PPG-based dipping estimates with the cuff-derived dipping values as the percentage of dipping values showing a concordant direction (dipping vs. non-dipping) between both devices, as well as the agreement on the amplitude of dipping in terms of bias and SD.

### Baseline case

The nocturnal dipping is an important variable to assess for cuff-less BP monitors as it is a way to evaluate their trending ability, i.e., their ability to track BP changes. Verifying this aspect is crucial, as recently highlighted by the European Society of Hypertension Working Group on BP Monitoring and Cardiovascular Variability^15^. Indeed, because their require to be calibrated, cuff-less devices could in theory simply output the BP value used for their calibration and may—in contexts where the BP is particularly stable—achieve artificially good accuracy^16^. Hence the importance to verify the ability of our approach to track the nocturnal BP dipping in the present study. Furthermore, we also compared our BP method to a so-called ‘baseline case’^16^. The baseline case is a naïve BP estimate that simply estimates the BP—for the whole 24-h period—as the BP value used for calibration. The baseline case is therefore expected to perform particularly poorly at night, as the calibration value is obtained from daytime measurements, and to be unable to track the nocturnal BP dipping properly.

### Device acceptance

To evaluate usability and comfort of the watch-like optical sensor, 44 participants filled a questionnaire. Wearing comfort, compatibility with daily activities, comparison to the conventional cuff-based device and side effects such as itching were evaluated.

## Results

The data of 8 participants were incomplete and therefore rejected prior to analysis (see upper part of Fig. 1): in 2 participants, the oscillometric cuff recording failed due to technical issues or low battery. In 5 participants, the PulseWatch recording was too short due to low battery and stopped before nighttime. In 1 participant, the PulseWatch detached after 7 h of recording, when the participant went to bed, and was not properly reattached afterwards. In the end, 70 recordings (19 with the PulseWatch placed at the upper arm) were available for data processing, with a median recording duration of 22.3 h per participant.

The characteristics of the 67 participants included in the final analysis are presented in Table 1. Half of study participants were healthy; the rest of participants were patients with known and treated for arterial hypertension or newly diagnosed with arterial hypertension.

**Table 1:** Biometric characteristics of the 67 participants included in the analysis. The data are given as “mean (SD) [range]” or “count (percentage)” over the cohort. *SD* standard deviation, *SBP*, *DBP* systolic, diastolic blood pressure.

Participant characteristics (n = 67)	Mean (SD) [range], or count (percentage)
Age (y)	42 (17) [20–84]
Height (cm)	173 (9) [153–190]
Weight (kg)	76 (14) [40–104]
Body mass index (kg/m^2^)	25.4 (4.0) [17.1–38.1]
Gender, male	41 (61.2)
Hypertension	32 (47.8)
Active smoking	6 (9.0)
Office SBP at rest, left arm (mmHg)	127 (16) [105–186]
Office DBP at rest, left arm (mmHg)	81 (11) [67–117]
Office SBP at rest, right arm (mmHg)	128 (15) [108–179]
Office DBP at rest, right arm (mmHg)	81 (11) [62–115]

**Figure 4:**
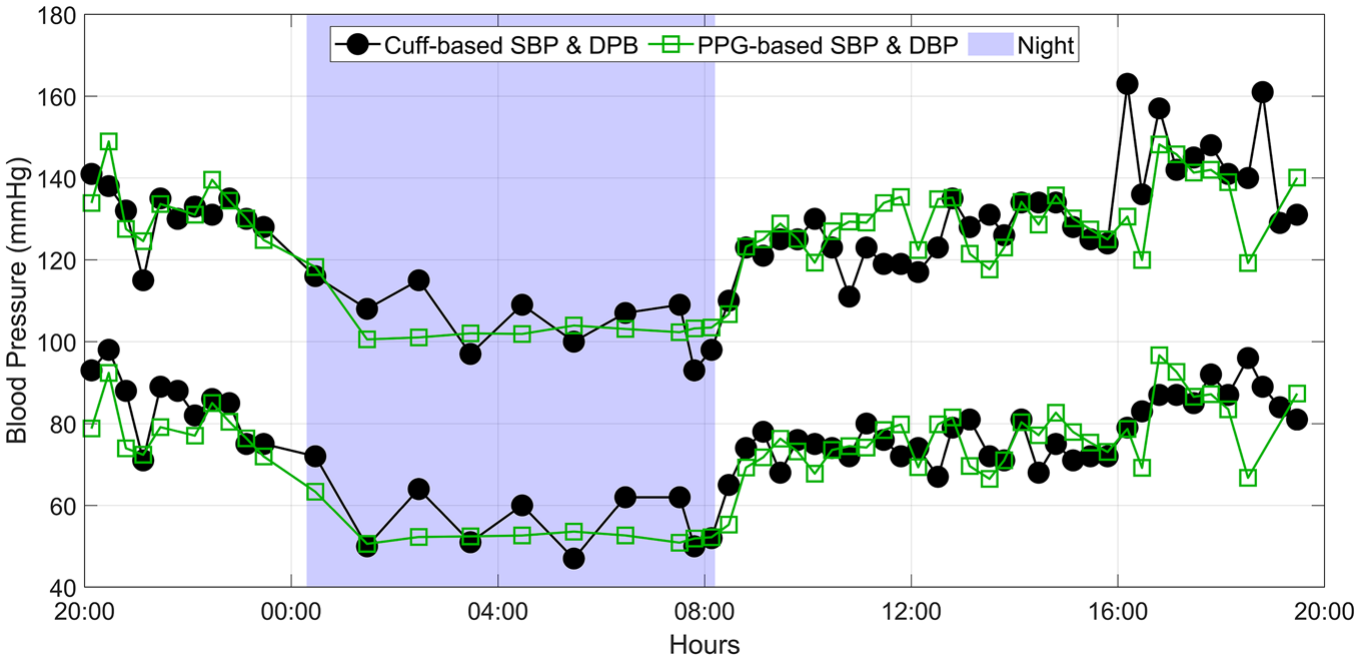
Example of a 24-h PPG-based BP profile compared to its cuff-based counterpart in a patient. *PPG* photoplethysmography, *SBP* systolic blood pressure, *DBP* diastolic blood pressure.

**Figure 5:**
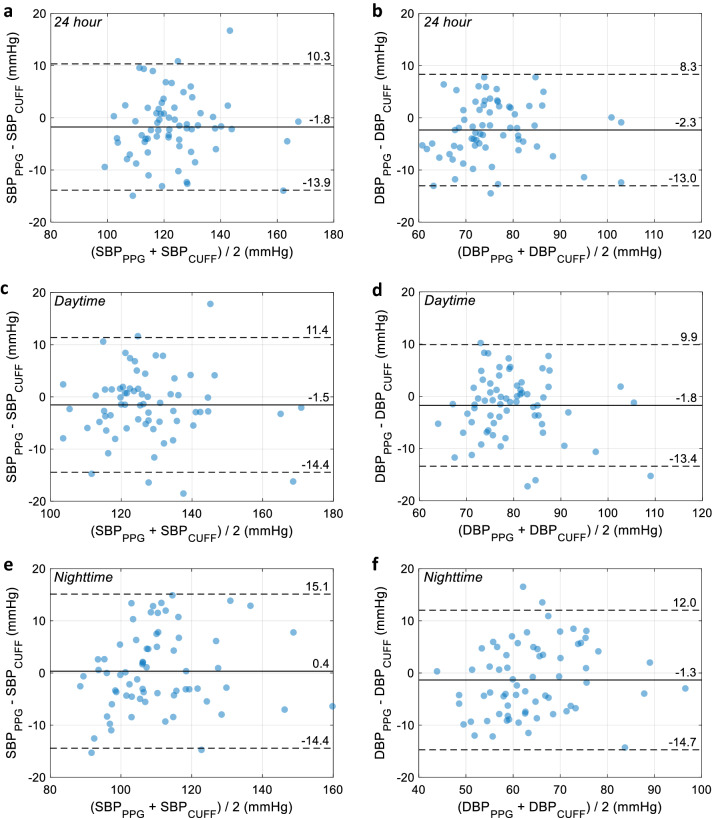
Bland–Altman plots comparing the PPG-derived SBP and DBP estimates to their cuff-based counterparts. (**a**,**b**) 24-h averages. (**c**,**d**) Daytime averages. (**e**,**f**) Nighttime averages. The solid lines depict the mean differences (biases), whereas the dashed lines depict the 95% limits of agreement. *PPG* photoplethysmography, *SBP* systolic blood pressure, *DBP* diastolic blood pressure.

For illustrative purposes, Fig. 4 shows an example of the 24-h cuff-based and PPG-based BP profile of a patient. In terms of cohort-wise agreement, Fig. 5 shows the Bland–Altman plots between the PPG-based cuff-less BP estimates and the cuff-derived BP values. The solid lines depict the mean difference (bias), whereas the dashed lines depict the 95% limits of agreement. The bias and SD between both methods—for the 24-h, daytime and nighttime values, and for all ABPM variables (including MBP, PP and HR)—are detailed in Table 2. In particular, we found 24-h, daytime, and nighttime average differences on SBP (bias ± SD) of − 1.8 ± 6.2 mmHg, − 1.5 ± 6.6 mmHg, and 0.4 ± 7.5 mmHg and on DBP of − 2.3 ± 5.4 mmHg, − 1.8 ± 5.9 mmHg, and − 1.3 ± 6.8 mmHg. All differences are normally distributed as assessed by the one-sample Kolmogorov–Smirnov test at the 5% significance level.

**Table 2:** Mean and standard deviation (SD) of the average 24-h, daytime and nighttime cuff-derived ABPM variables and their PPG-derived counterparts. *ABPM* ambulatory blood pressure monitoring, *PPG* photoplethysmography, *SBP*, *MBP*, *DBP* systolic, mean, diastolic blood pressure, *PP* pulse pressure, *HR* heart rate.

Mean ± SD over n = 67 participants	ABPM variable				
	SBP (mmHg)	DBP (mmHg)	MBP (mmHg)	PP (mmHg)	HR (bpm)
24-h					
Cuff	124.0 ± 13.8	76.9 ± 9.1	92.1 ± 9.3	47.1 ± 8.8	72.0 ± 10.4
PPG	122.2 ± 14.0	74.6 ± 9.4	89.8 ± 10.0	47.7 ± 8.8	70.8 ± 9.7
PPG—Cuff	− 1.8 ± 6.2*	− 2.3 ± 5.4*	− 2.2 ± 5.8*	0.6 ± 3.5	− 1.2 ± 1.8*
Daytime					
Cuff	128.2 ± 13.9	80.5 ± 9.3	95.6 ± 9.4	47.7 ± 9.2	75.3 ± 10.8
PPG	126.6 ± 13.6	78.7 ± 8.7	94.1 ± 9.3	47.9 ± 8.7	74.1 ± 10.1
PPG—Cuff	− 1.5 ± 6.6	− 1.8 ± 5.9*	− 1.5 ± 6.2*	0.2 ± 3.9	− 1.3 ± 2.1*
Nighttime					
Cuff	110.1 ± 14.0	64.6 ± 10.0	80.2 ± 10.0	45.5 ± 8.4	62.8 ± 9.1
PPG	110.5 ± 15.0	63.3 ± 11.2	78.4 ± 11.6	47.2 ± 9.0	61.6 ± 8.9
PPG—Cuff	0.4 ± 7.5	− 1.3 ± 6.8	− 1.8 ± 7.0*	1.7 ± 5.4*	− 1.2 ± 3.0*

*Paired-sample *t* test: Significant difference at the 5% significance level (*P* < 0.05).

**Figure 6:**
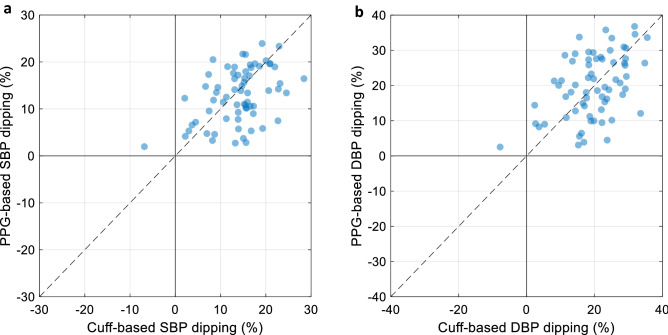
Four-quadrant plots comparing the PPG-derived nocturnal dipping estimates to their cuff-based counterparts for SBP (**a**) and DBP (**b**). *PPG* photoplethysmography, *SBP* systolic blood pressure, *DBP* diastolic blood pressure.

Figure 6 shows a four-quadrant plot depicting the relationship between the nocturnal dipping values as estimated by both methods. The CR on the estimated direction of dipping between both methods was of 98.5% for both SBP and DBP. The average (± SD) difference in dipping amplitude estimation was of − 1.2% (± 6.3%) for SBP, and 0.2% (± 9.0%) for DBP. The median per-subject nocturnal SBP and DBP dipping values were 15.0% and 19.8% for the cuff, respectively, and 13.4% and 20.0% for the PPG. On the cuff measurements, the median per-subject 95% confidence intervals (CI) on the nocturnal SBP and DBP dipping values were of 11.7% and 15.2%, respectively. For the PPG, smaller 95% CI were found (8.9% and 12.4% for SBP and DBP, respectively).

The results obtained with the baseline case—the naïve BP estimate that uses the calibration value as BP estimate for the whole 24-h period—are provided in the Supplementary Information, and especially in Supplementary Figs. S1, S2 and S3, and in Supplementary Table S1. In short, we obtained with the baseline case 24-h, daytime, and nighttime average differences on SBP of − 2.3 ± 6.9 mmHg, − 6.5 ± 7.2 mmHg, and 11.5 ± 8.4 mmHg and on DBP of − 2.9 ± 6.2 mmHg, − 6.5 ± 6.5 mmHg, and 9.4 ± 7.3 mmHg. No concordance (CR = 0%) was found between the nocturnal dipping values estimated by the baseline case and by the brachial cuff.

The detailed results of the device acceptance questionnaire are provided in Supplementary Table S2. The results show that the watch-like device was comfortable to wear, highly compatible with daily activities and, in comparison to the standard ABPM, was evaluated to be better. Only three participants reported slight local itching and one participant skin redness.

Finally, the PPG data acceptance rate represents the percentage of the time where the PPG signal is usable for BP estimation. We found a median (first and third quartiles) per-patient PPG acceptance rate of 89% (69–96%) during the day, and 100% (100–100%) at night. In the 48 patients for whom the sensor was placed at the wrist, the data acceptance rate was 89% (65–93%) during the day, and 100% (100–100%) at night. For the 19 patients for whom it was placed at the upper arm, where PPG was hypothesized to be less sensitive to arm motion artefacts, the data acceptance rate was 96% (87–100%) during the day, and 100% (100–100%) at night.

## Discussion

The purpose of the present study was to investigate the feasibility of using a PPG-based cuff-less BP estimation approach for 24-h ABPM by comparing it to the conventional oscillometric brachial cuff. We found good agreement (low bias and SD) between the PPG-based and the cuff-derived BP values for 24-h, daytime and nighttime averages. Despite comparing our cuff-less approach to an oscillometric device, the latter having its own inaccuracy and imprecision, the differences between both devices remained within the limits recommended by the ISO 81060-2 international standard when comparing a device to gold-standard references^17^, with biases within ± 5 mmHg and SD values ≤ 8 mmHg. We also compared the ability of the PPG-based measurement to estimate the nocturnal BP dipping. Excellent concordance (98.5%) was found between both methods when assessing the direction of dipping. Evaluating the agreement in terms of amplitude of dipping showed small biases, but relatively large variability (SD of 6.3% and 9.0% for SBP and DBP, respectively). A part of this variability may be due to the intrinsic uncertainty associated with the calculation of the dipping ratio, as it is calculated using two other variables, namely the daytime and nighttime BP averages, both of which have their own uncertainty, making the 95% CI of their ratio even larger. Over the entire cohort of patients, the per-patient 95% CI of the nocturnal SBP dipping value was of 11.7% and was even larger (15.2%) for DBP. This puts into perspective the relatively high imprecision (high SD) found on the differences between the cuff-based and PPG-based dipping estimates, even more so that the ranges of the 95% CI on the PPG-based SBP and DBP dipping values (8.9% and 12.4%, respectively) were found to be ~ 27% smaller than their cuff-based counterparts. This suggests that a significant proportion of the disagreement between both devices in terms of dipping estimation may actually be due to the cuff’s precision (the validation study of the Spacelabs 90227 OnTrak device reports a SD of 7 mmHg for both SBP and DBP when compared to auscultatory measurements^7^). This may also explain why the SD of the differences between the PPG-based and the cuff-derived BP values was found to be slightly higher at night (Table 2).

Other PPG-based approaches have recently been evaluated for cuff-less 24-h ABPM and have failed to produce positive results, especially in terms of trending ability, when trying to track the daytime to nighttime BP dipping^18,19^. This is in line with the recent emphasis placed by hypertension expert groups on the potential shortcomings of cuff-less approaches and the importance of demonstrating trending ability^15^. In the present method-comparison study, we have been able to demonstrate trending ability through a high concordance (98.5%) and low biases (≤ 1.2%) on the dipping estimates, along with low biases (≤ 2.3 mmHg) on the 24-h, daytime and nighttime BP values. This difference with other PPG-based approaches may be due to the fact that our PWA algorithm was specifically trained to track BP changes rather than estimating absolute BP values^10^. This added value of our approach was further highlighted by the lower performance of the baseline case in our study not only in terms of SD values, but most markedly on the nighttime biases (11.5 and 9.4 mmHg on SBP and DBP, respectively) and inexistent trending ability (CR = 0%).

An important aspect to point out is the data acceptance rate of our proposed PPG-based approach. Out of 70 patients, 3 had to be rejected prior to statistical analysis due to insufficient amount of high-quality PPG signals. In the remaining 67 patients, the median per-patient PPG acceptance rate (time coverage) was 89% during the day, and 100% at night. Several factors may come into play in explaining the 11% daytime rejection rate. Excessive motion during daily activities induces motion artefacts that distort the morphology of the PPG signals, and is by far the main cause of PPG data rejection during daytime. This seems to be confirmed by the difference in acceptance rate we found between the patients for whom the sensor was placed at the wrist (daytime acceptance rate of 89%) compared to those for whom the sensor was placed at the upper arm (daytime acceptance rate of 96%), where the amplitude of arm motion is smaller and therefore has a lower influence on the PPG data. Insufficient tightening of the sensor is another possible cause, leading to poor sensor-skin interface and therefore noisy PPG signals. On the other hand, excessive tightening may also decrease signal amplitude, by decreasing local perfusion. Low tissue perfusion itself, irrespective of its cause, tends to affect the signal-to-noise ratio of PPG signals, a well-known issue in pulse oximetry^20^. Lastly, the PPG signals in our study were acquired at the wrist and upper arm, i.e., using PPG in reflectance mode (by opposition to transmission mode). Transmission mode PPG is commonly used by standard pulse oximeters, but its usage is limited to body extremities (e.g., fingertip), as the light needs to go through the pulsatile tissue. On the other hand, reflectance mode PPG can be used at virtually any body location—making its 24-h use less obtrusive—but relies on back-scattered light, of much lower amplitude. Reflectance PPG signals have therefore lower signal-to-noise ratios.

In terms of device obtrusiveness, the results of the device acceptance questionnaire revealed that a watch-like device worn at the wrist or upper arm was found to be more comfortable, highly compatible with daily activities and was overwhelmingly preferred to its cuff-based alternative by the participants.

Our study was designed as a method-comparison feasibility study rather than a clinical validation. Several study limitations should be addressed in a future investigation for a formal validation of the approach. In particular, the possibility to perform the calibration procedure directly at the doctor’s office should be implemented through real-time control of the raw PPG signal quality during the procedure. Particular focus should be brought on ensuring very controlled conditions and excellent PPG data quality during the procedure, as any bias introduced by the cuff at that point will be passed on all PPG-based estimates during the 24-h ABPM session. Increasing the frequency of cuff-based measurements at nighttime to two readings per hour—as recommended by the ABPM practice guidelines of the European Society of Hypertension^2^—should allow improving the comparability of the nighttime BP values between both methods. Finally, the interchangeability of our PPG-based approach compared to the conventional brachial cuff should be formally evaluated in terms of hypertension diagnostic ability.

Our study shows encouraging results suggesting that 24-h ABPM using a cuff-less watch-like device at the wrist or upper arm may indeed be possible and eventually make its way into clinical practice, provided that proper validation studies confirm its clinical potential. Due to its cuff-less nature, the PPG-based measurement is less obtrusive than its cuff-based counterpart—as no artery occlusion is necessary—making the measurement unnoticeable and painless, with an expected significant improvement in terms of patient comfort and sleep quality. Further investigations are needed to properly validate the approach for 24-h ABPM, particularly regarding the impact of the calibration procedure when performed at the doctor’s office. Real-time automatic evaluation of the PPG data quality by the sensor with direct feedback to the physician—necessary for a proper calibration procedure—is also expected to improve data acceptance, by allowing the optimization of the attachment (tightness, placement, etc.) of the watch-like sensor. Alternatively, to avoid any risk of low daytime PPG data acceptance rate, a hybrid approach combining cuff-based monitoring during the day and cuff-less PPG-based monitoring at night could easily be implemented, and already provide a significant improvement in terms of patient comfort compared to the current solution.

## Supplementary Information

Supplementary Information.

## Data Availability

The data that support the findings of this study are available from the corresponding author on reasonable request.
