# Supplementary material for: Method-comparison study between a watch-like sensor and a cuff-based device for 24-h ambulatory blood pressure monitoring
Source: Sci Rep. 2023 Apr 15;13:6149. doi: 10.1038/s41598-023-33205-z (PMC10105693; doi:10.1038/s41598-023-33205-z)
Supplement: Supplementary file 1 — Supplementary Information. [file 41598_2023_33205_MOESM1_ESM.pdf]

Supplementary Information for “Method-comparison study between a watch-like sensor and a cuff-based device for 24-hour ambulatory blood pressure monitoring” by Proença et al.

1. Patient eligibility, inclusion, and exclusion criteria

Eligibility Criteria

- Ages Eligible for Study: 18 Years and older (Adult, Older Adult)
- Sexes Eligible for Study: All
- Accepts Healthy Volunteers: Yes

Inclusion Criteria

- Patients > 18y
- Good understanding of written and oral German
- Signed informed consent
- Scheduled for 24h blood pressure exam

Exclusion Criteria

- Patients with unhealthy mental state
- Patients with an active implantable medical device (AIMD)
- Arteriopathy of the upper limbs with/without stenosis
- Severe congestive heart failure (LV-EF ≤25%)
- Sever aortic-valve stenosis mean gradient >40mmHg, valve area <1cm2)
- Congenital heart disease including aortic isthmus stenosis
- Untreated heart rhythm disorders, heart rate at rest > 120/bpm
- Severe untreated arterial hypertension (BPsyst > 180mmHg, BPdiast > 100mmHg)
- Atrial fibrillation
- Instable angina pectoris
- Malcompliance concerning medication intake
- Active alcohol or drug abuse
- Pregnancy or lactation (women of childbearing age will be asked to performed urinary pregnancy test before the screening phase)

2. Results on the baseline case

For illustrative purposes, Supplementary Fig. S1 shows the 24-hour cuff-based and baseline case-based BP profile of the same patient shown in Fig. 4 of the main article. In terms of cohort-wise agreement, Supplementary Fig. S2 shows the Bland-Altman plots between the baseline case-based cuff-less BP estimates and the cuff-derived BP values. The bias and SD between both methods – for the 24-hour, daytime and nighttime values, and for all ABPM variables (including MBP, PP and HR) – are detailed in Supplementary Table S1. In particular, we find 24-hour, daytime, and nighttime average differences on SBP (bias ± SD) of -2.3 ± 6.9 mmHg, -6.5 ± 7.2 mmHg, and 11.5 ± 8.4 mmHg and on DBP of -2.9 ± 6.2 mmHg, -6.5 ± 6.5 mmHg, and 9.4 ± 7.3 mmHg. All differences are normally distributed as assessed by the one-sample Kolmogorov-Smirnov test at the 5% significance level.

Supplementary Fig. S3 shows a four-quadrant plot depicting the relationship between the nocturnal dipping values as estimated by both methods. By design, the baseline case is unable to track the nocturnal dipping. The CR on the estimated direction of dipping is therefore 0% for both SBP and DBP. The average (±SD) difference in dipping amplitude estimation is of -14.0% (±6.1%) for SBP, and -19.7% (±8.4%) for DBP.

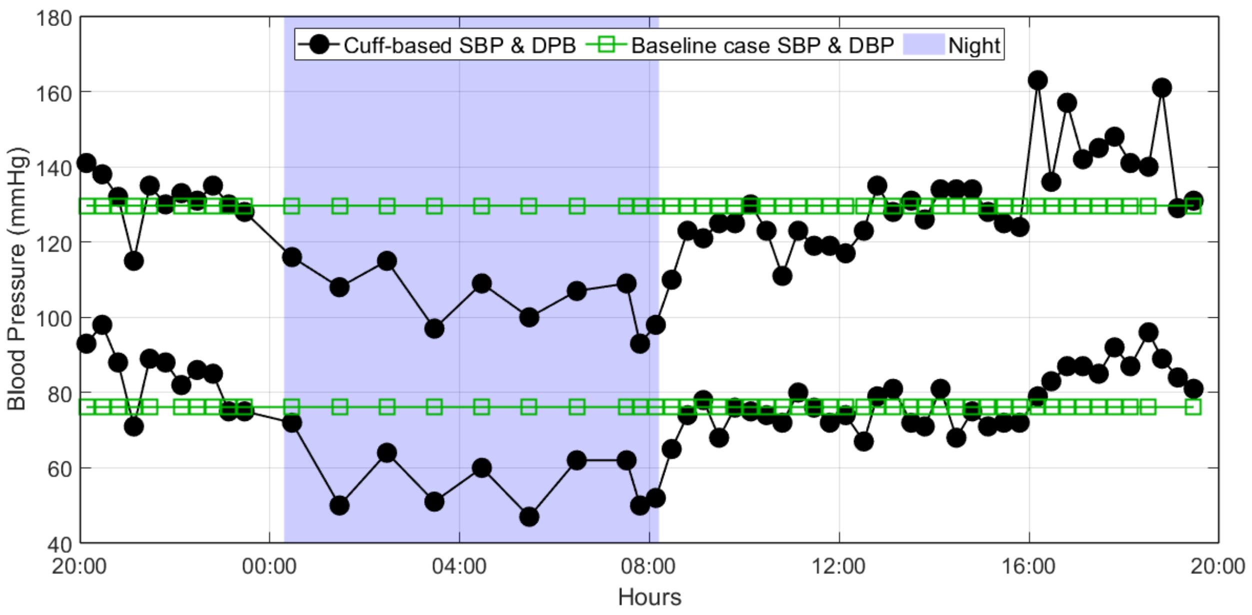

Supplementary Figure S1: Example of a 24-hour baseline case BP profile compared to its cuff-based counterpart in a patient. SBP: systolic blood pressure; DBP: diastolic blood pressure.

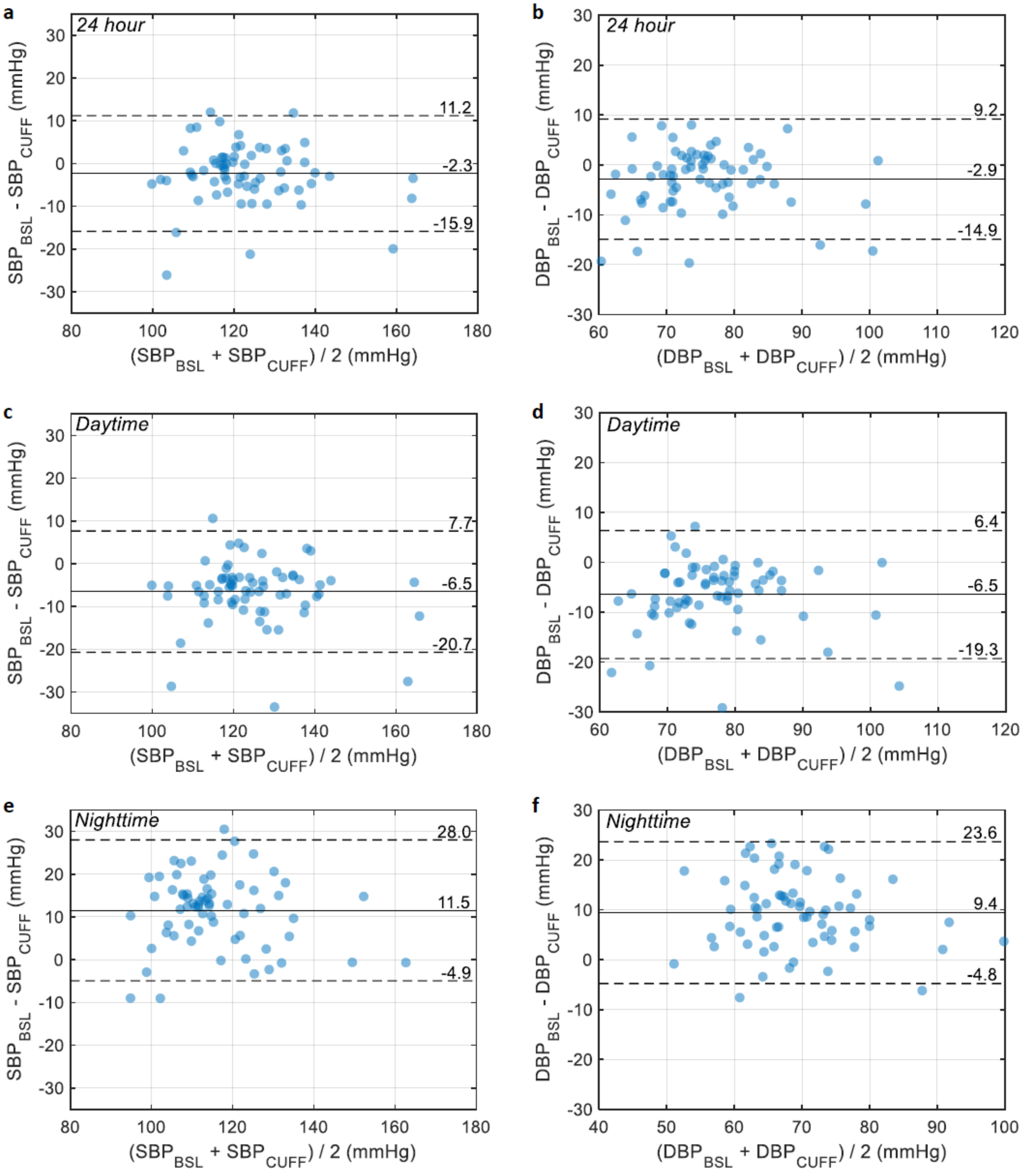

Supplementary Figure S2: Bland-Altman plots comparing the baseline case-derived SBP and DBP estimates to their cuff-based counterparts. Panels a and b: 24-hour averages. Panels c and d: Daytime averages. Panels e and f: Nighttime averages. The solid lines depict the mean differences (biases), whereas the dashed lines depict the 95% limits of agreement. BSL: baseline case; SBP: systolic blood pressure; DBP: diastolic blood pressure.

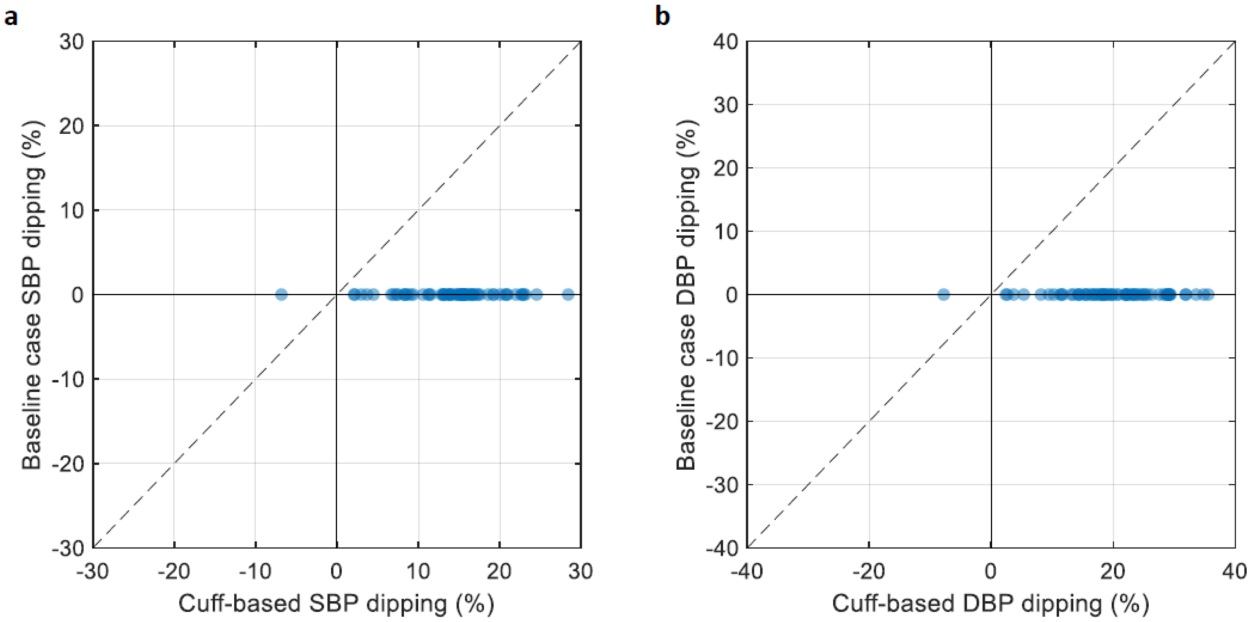

Supplementary Figure S3: Four-quadrant plots comparing the baseline case-derived nocturnal dipping estimates to their cuff-based counterparts for SBP (panel a) and DBP (panel b). SBP: systolic blood pressure; DBP: diastolic blood pressure.

Supplementary Table S1: Mean and standard deviation (SD) of the average 24-hour, daytime and nighttime cuff-derived ABPM variables and their baseline case-derived counterparts. ABPM: ambulatory blood pressure monitoring; SBP, MBP, DBP: systolic, mean, diastolic blood pressure; PP: pulse pressure; HR: heart rate.

Mean ± SD over n=67 participants		ABPM variable				
		SBP (mmHg)	DBP (mmHg)	MBP (mmHg)	PP (mmHg)	HR (bpm)
24-hour						
	Cuff	124.0 ± 13.8	76.9 ± 9.1	92.1 ± 9.3	47.1 ± 8.8	72.0 ± 10.4
	Baseline case	121.7 ± 13.2	74.0 ± 9.1	89.3 ± 9.3	47.6 ± 8.6	69.1 ± 9.3
	Baseline case – Cuff	-2.3 ± 6.9 *	-2.9 ± 6.2 *	-2.8 ± 6.1 *	0.5 ± 3.3	-3.0 ± 5.8 *
Daytime						
	Cuff	128.2 ± 13.9	80.5 ± 9.3	95.6 ± 9.4	47.7 ± 9.2	75.3 ± 10.8
	Baseline case	121.7 ± 13.2	74.0 ± 9.1	89.3 ± 9.3	47.6 ± 8.6	69.1 ± 9.3
	Baseline case – Cuff	-6.5 ± 7.2 *	-6.5 ± 6.5 *	-6.3 ± 6.6 *	-0.0 ± 3.6	-6.3 ± 5.6 *
Nighttime						
	Cuff	110.1 ± 14.0	64.6 ± 10.0	80.2 ± 10.0	45.5 ± 8.4	62.8 ± 9.1
	Baseline case	121.7 ± 13.2	74.0 ± 9.1	89.3 ± 9.3	47.6 ± 8.6	69.1 ± 9.3
	Baseline case – Cuff	11.5 ± 8.4 *	9.4 ± 7.3 *	9.1 ± 7.2 *	2.1 ± 4.9 *	6.3 ± 6.4 *

* Paired-sample *t*-test: Significant difference at the 5% significance level (*P*<0.05).

3. Device acceptance questionnaire results on 44 participants

Supplementary Table S2: Device acceptance results on 44 participants in terms of (a) wearing comfort, (b) compatibility with daily activities, (c) comparison to conventional 24-hour BP device, and (d) side effects.

Participant number	Wearing comfort	Compatibility with daily activities	Comparison to conventional 24-hour BP device	Side effects (skin redness, itching, pain)
	Comfortable (0 pt)	*No limitations (0 pt)*	*Better (-1 pt)*	*No (0 pt)*
	Neutral (1 pt)	*Slightly limiting (1 pt)*	*Equal (0 pt)*	
	Slightly uncomfortable (2 pts)	*Highly limiting (2 pts)*	*Worst (1 pt)*	*Yes (3 pts)*
	Highly uncomfortable (3 pts)	*Incompatible (3 pts)*		
1	0	0	-1	0
2	0	0	-1	0
3	1	0	-1	3 (skin redness)
4	2	1	-1	3 (itching, slight pain)
5	0	1	-1	0
6	2	0	-1	0
7	3	0	1	3 (itching)
8	0	0	-1	0
9	0	0	0	0
10	0	0	-1	0
11	0	0	-1	0
12	2	1	-1	3 (itching)
13	0	0	-1	0
14	0	0	-1	0
15	1	0	-1	0
16	1	1	-1	0
17	2	1	-1	0
18	1	0	-1	0
19	0	0	-1	0
20	1	0	-1	0

21	1	0	-1	0
22	1	0	-1	0
23	0	0	-1	0
24	1	0	-1	0
25	1	1	0	0
26	0	0	-1	0
27	0	0	-1	0
28	1	1	-1	0
29	0	0	-1	0
30	0	0	-1	0
31	0	0	-1	0
32	0	0	-1	0
33	1	0	0	0
34	1	0	0	0
35	0	0	-1	0
36	0	1	-1	0
37	0	0	-1	0
38	0	1	-1	0
39	0	0	-1	0
40	0	0	-1	0
41	0	0	-1	0
42	1	0	-1	0
43	1	0	-1	0
44	0	0	-1	0
**Mean**	**0.57**	**0.20**	**-0.86**	**0.27**
**SD**	**0.76**	**0.41**	**0.41**	**0.87**
